# Effects of PDE-5 Inhibition on the Cardiopulmonary System After 2 or 4 Weeks of Chronic Hypoxia

**DOI:** 10.1007/s10557-019-06887-9

**Published:** 2019-07-01

**Authors:** Coline Nydegger, Antonio F. Corno, Ludwig K. von Segesser, Maurice Beghetti, Michele Samaja, Giuseppina Milano

**Affiliations:** 1 Department Cœur-Vaisseaux, Cardiac Surgery center, University Hospital of Lausanne, Lausanne, Switzerland; 2 Cardiovascular Research Center, University of Leicester, Leicester, UK; 3 Cardiovascular Research Unit, University Hospital of Lausanne, Lausanne, Switzerland; 4 Pediatric Cardiology Unit, University of Geneva, Geneva, Switzerland; 5 Centre Universitaire Romand de Cardiologie et Chirurgie Cardiaque Pédiatrique, Children’s University Hospitals, Geneva and Lausanne, Lausanne, Switzerland; 6 Department of Health Science, University of Milan, Milan, Italy

**Keywords:** Chronic hypoxia, Adaptation, Phosphodiesterase-5 inhibition, Pulmonary angiogenesis, Right-ventricle hypertrophy

## Abstract

**Purpose:**

In pulmonary hypertension (PH), hypoxia represents both an outcome and a cause of exacerbation. We addressed the question whether hypoxia adaptation might affect the mechanisms underlying PH alleviation through phosphodiesterase-5 (PDE5) inhibition.

**Methods:**

Eight-week-old male Sprague-Dawley rats were divided into two groups depending on treatment (placebo or sildenafil, a drug inhibiting PDE5) and were exposed to hypoxia (10% O_2_) for 0 (*t*0, *n* = 9/10), 2 (*t*2, *n* = 5/5) or 4 (*t*4, *n* = 5/5) weeks. The rats were treated (0.3 mL i.p.) with either saline or sildenafil (1.4 mg/Kg per day).

**Results:**

Two-week hypoxia changed the body weight (− 31% vs. − 27%, respectively, *P* = NS), blood hemoglobin (+ 25% vs. + 27%, *P* = NS) and nitrates+nitrites (+ 175% vs. + 261%, *P* = 0.007), right ventricle fibrosis (+ 814% vs. + 317%, *P* < 0.0001), right ventricle hypertrophy (+ 84% vs. + 49%, *P* = 0.007) and systolic pressure (+ 108% vs. + 41%, *P* = 0.001), pulmonary vessel density (+ 61% vs. + 46%, *P* = NS), and the frequency of small (< 50 µm wall thickness) vessels (+ 35% vs. + 13%, *P* = 0.0001). Most of these changes were maintained for 4-week hypoxia, except blood hemoglobin and right ventricle hypertrophy that continued increasing (+ 52% vs. + 42%, *P* = NS; and + 104% vs. + 83%, *P* = 0.04). To further assess these observations, small vessel frequency was found to be linearly related with the right ventricle-developed pressure independent of hypoxia duration.

**Conclusions:**

Thus, although hypoxia adaptation is not yet accomplished after 4 weeks, PH alleviation by PDE5 inhibition might nevertheless provide an efficient strategy for the management of this disease.

## Introduction

The pathophysiology of pulmonary hypertension (PH) includes progressive rising of the pulmonary artery pressure, with subsequent straining of the right ventricle (RV) thereby causing hypertrophy and leading to potential right heart failure and/or ventricular arrhythmias. In addition to the fact that PH contributes to the impairment of the pulmonary function with subsequent deterioration of the gas exchange, thereby causing systemic hypoxia, hypoxia per se also represents one of the main complications of PH, establishing an irreversible vicious circle, whereby PH causes hypoxia, which in turn exacerbates PH [1]. Healthy individuals, however, may in part compensate systemic hypoxia through adaptation, a complex phenomenon recruiting the pulmonary, cardiovascular, hematological, metabolic, and endocrine systems [2, 3]. Is the mechanism of hypoxia adaptation also applicable in patients with PH, caused either by congenital heart defects or by acquired diseases? This question might be relevant because a therapy proven to be effective in the treatment of PH, e.g., phosphodiesterase-5 (PDE5) inhibition by sildenafil [4, 5], generally used as a vasodilator agent to antagonize the vasoconstriction present in pulmonary hypertension, could be extended to treat also the negative effects of pulmonary hypertension such as subsequent hypoxia. Therefore, it is important to assess whether PH-induced hypoxia is also responding to adaptation. Remarkably, while studies have been reported on the long-term effects in the pediatric population with PH [6–9], evidence for similar long-term beneficial effects of PDE5 inhibitors in COPD patients is still controversial [10–12], thereby justifying experimental studies aiming to answer the question whether the effects of hypoxia, and hence of PDE5 inhibition, are time-dependent.

The design of the present study was focused on assessing if some of the mechanisms known to underlie alleviation of hypoxia-induced PH by PDE5 inhibition [13] are time-dependent. Such information might be important not only to confirm the viability of the mechanisms for a better treatment of hypoxia of longer duration but also to assess whether other time-dependent mechanisms, potentially linked to hypoxia adaptation, may eventually override the protection afforded by sildenafil and introduce bias in the interpretation of results. Therefore, the target of this study was to test whether the cardiopulmonary dysfunction caused by hypoxia and the correction provided by sildenafil display time-dependent features. To this purpose, we examined two time durations (2 and 4 weeks) of challenge (hypoxia) and treatment (sildenafil).

## Methods

### Protocol

We used male 8-week old Sprague-Dawley rats (200–250 g initial nominal weight). Rats were randomly divided into two groups depending on treatment (without/with sildenafil) and were exposed to hypoxia (10% O_2_) as described previously [13] for 0 (*t*0, *n* = 9/10), 2 (*t*2, *n* = 5/5), or 4 (*t*4, n = 5/5) weeks. Rats were treated (0.3 mL i.p.) with either saline or sildenafil (1.4 mg/kg/day). Treatments started in correspondence to exposure to hypoxia.

Twenty-four hours after the last treatment, rats were anesthetized (80 mg/kg xylazine, 100 mg/kg ketasol, and 1500 IU heparin i.p.) in the compensation chamber at 10% O_2_.

To assess the hemodynamics, anesthetized rats were placed on a heating pad at 37 °C and ventilated at 50 cycles min^−1^ (tidal volume of 2.5 mL, Harvard Apparatus model 683, Holliston, MA, USA) with either room air (21% O_2_) or hypoxic (10% O_2_) atmosphere for normoxic and hypoxic groups, respectively. To evaluate the LV pressures, a Millar catheter was introduced into the LV via the right carotid artery. To evaluate RV pressures, the chest cavity was opened and Millar catheter was directly introduced in the RV cavity using a 24-gauge needle, and connected to a MPVS Ultra system (Millar Instruments) to record the pressure waves. At the end of hemodynamic analysis, lungs and hearts were perfused en-block with PBS via the RV with efflux through a small opening in the left atrium [14], placed in PBS and kept on ice. Atria were removed, the RV and the septum (S) were isolated, blotted dry and weighed to obtain the RV/(LV + S) ratio.

### Pulmonary Vascular Remodeling

The degree of muscularization of pulmonary arterioles was assessed from immunohistochemical staining of the small pulmonary artery, as previously described [13]. Briefly, the lungs were inflated with 10% formalin at 25 cm H_2_O pressure through the trachea for 15 min, excised and finally processed for paraffin embedding. Paraffin-embedded lungs were serially sectioned at 8-μm thickness. Following citrate-based antigen retrieval, the sections were blocked with 5% (*v*/*v*) goat serum for 1 h. Then, sections were incubated with an antibody against smooth muscle α-actin (α-SMA 1:250, clone 1A4, Sigma-Aldrich) overnight at 4 °C, followed by a goat anti-mouse IgG secondary antibody (1/500, DAKO), developed with 3,3′-diaminobenzidine, and counter stained with hematoxylin and eosin. All transversal cut arterioles were quantitatively analyzed at × 40 magnification using an images analysis system Nikon eclipse 80i camera and NIH image software (Nikon Instruments Inc., Melville, NY, USA). Pulmonary arterial thickening was assessed by calculating the percent pulmonary artery thickness using the following formula: 100 × (perivascular area-luminal area)/luminal area. Pulmonary arteries were categorized as follows: < 50 μm, 50–100 μm, 100–200 μm, and > 200 μm. Ten vessels were analyzed for each rat, in six rats per group per time-point. Morphological analyses were conducted in a double-blind method.

### RV Fibrosis

RV fibrosis was evaluated by routine Masson’s staining. The images were taken in random field at a magnification of × 200, and the area of cardiac fibrosis was calculated by using NIH image software.

### Biochemical Measurements

Standard Western blotting analysis was performed in lung and cardiac lysates [14] using antibodies against p-eNOS (Ser^1177^, 1:1000, Cell Signaling Technology) and e-NOS (1:1000, Santa Cruz Biotechnology, Santa Cruz). Secondary HRP-conjugated antibodies were applied for 1 h at room temperature, and signals were detected by using the enhanced chemiluminescence system (Amersham, Arlington Heights, IL, USA) of a commercial ECL kit. Quantification of the band intensities was performed using NIH ImageJ.

Blood hemoglobin (Hb, Drabkin’s reagent) and plasma nitrates and nitrites (NOx, colorimetric Griess reaction) were measured in heparinized samples taken after euthanasia.

### Statistics

Data are expressed as mean ± SEM. To measure the effect of hypoxia duration, we performed one-way analysis of variance followed by the Bonferroni test to detect significant differences between the time points. To measure the differences between the two treatments at every time point, we used the unpaired *t* test. The significance level was set at *P* = 0.05.

## Results

**Fig. 1:**
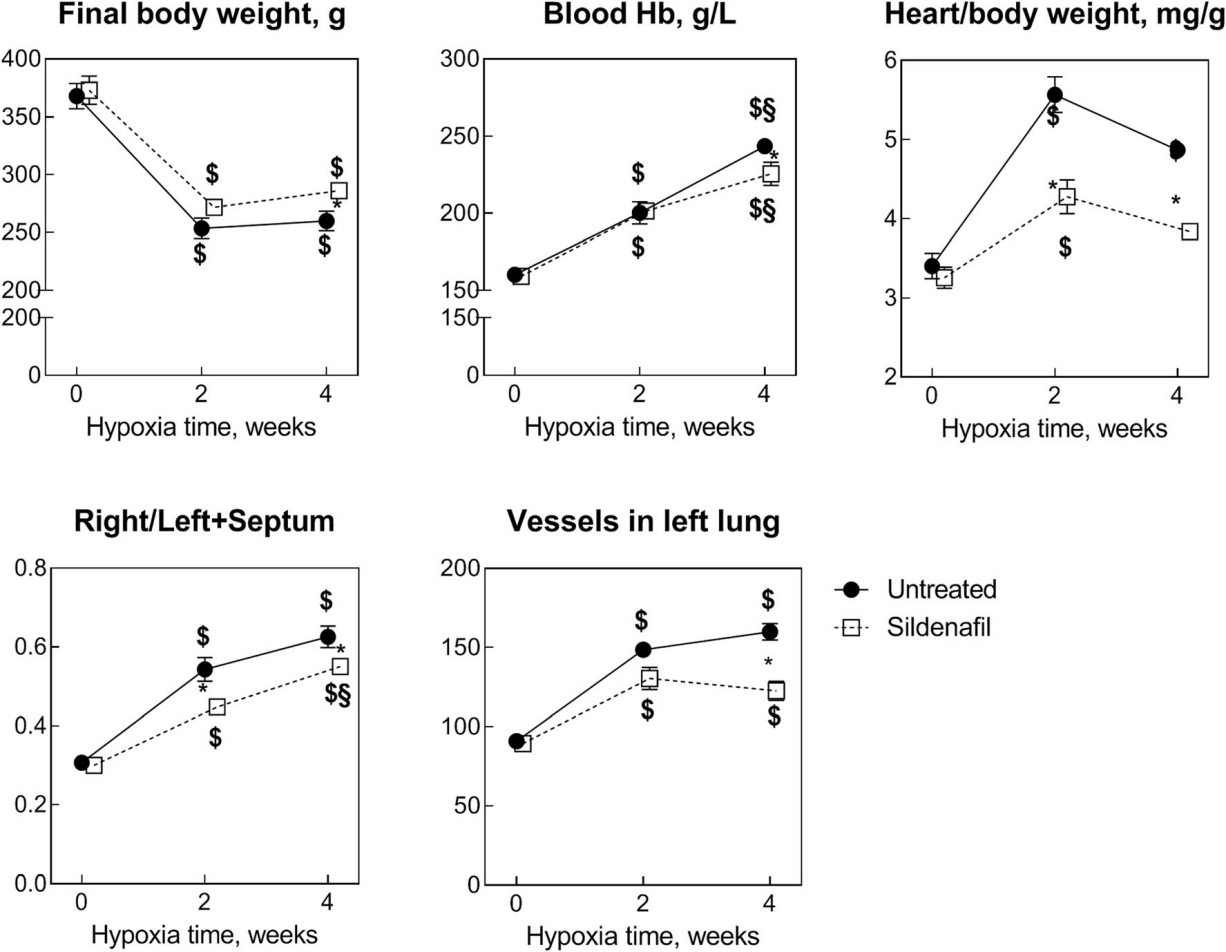
Effects of various durations of hypoxia in the absence (filled circles) and in the presence (empty squares) of sildenafil (1.4 mg/kg/day). The vertical bars represent the SEM. Sildenafil data points are slightly nudged to the right to enable distinguishing overlapping symbols and error bars. $,§*P* < 0.05 (ANOVA and Bonferroni tests) with respect to *t*0 and *t*2, respectively. *P < 0.05 (unpaired Student’s *t* test) between control and sildenafil groups for each time point

All the animals used in this study survived the protocols. Hypoxia markedly decreased the body weight at *t*2 irrespectively of sildenafil treatment (*P* < 0.05 vs. *t*0 for both control and sildenafil, Fig. 1). This change was maintained at *t*4 (*P* = NS vs. *t*2 in both groups). A slight favorable effect of sildenafil was, however, noticed at *t*4 (*P* = 0.046). Hypoxia steadily increased blood [Hb] at both *t*2 (*P* < 0.05 vs. *t* = 0) and *t*4 (*P* < 0.05 vs. *t* = 2 weeks). A slight borderline effect of sildenafil was observed at *t*4 (*P* = 0.052).

At *t*2, the heart/body weight ratio increased by 60% in controls (*P* < 0.05 vs. *t*0) and did not change further at *t*4 (*P* = NS vs. *t* = 2 weeks), indicating no increase in myocardial hypertrophy after 2-week hypoxia. Sildenafil halved the progression of myocardial hypertrophy at both times (*P* = 0.0035 and *P* < 0.00001 vs. control at *t*2 and *t*4, respectively). To follow up the effect on RV hypertrophy, we used the RV/(LV + S) ratio. This index increased markedly at *t*2 (*P* < 0.05 vs. *t*0 for both control and sildenafil), but the increase was less marked in sildenafil than controls (*P* = 0.007). At *t*4, the RV/(LV + S) ratio did not change appreciably in control hearts (*P* = NS vs. *t*2) but increased significantly in sildenafil hearts (*P* < 0.05 vs *t*2). Consequently, the difference between control and sildenafil decreased but remained significant (*P* = 0.046).

Two-week hypoxia augmented the vessel density in both the right and left lungs (only the left lung is shown for clarity, *P* < 0.05 vs. *t*0). Only a borderline (*P* = 0.058) effect of sildenafil was observed. At *t*4, the vessel density did not change appreciably vs. *t*2, but the anti-angiogenic effect of sildenafil became very clear (*P* < 0.0001).

**Fig. 2:**
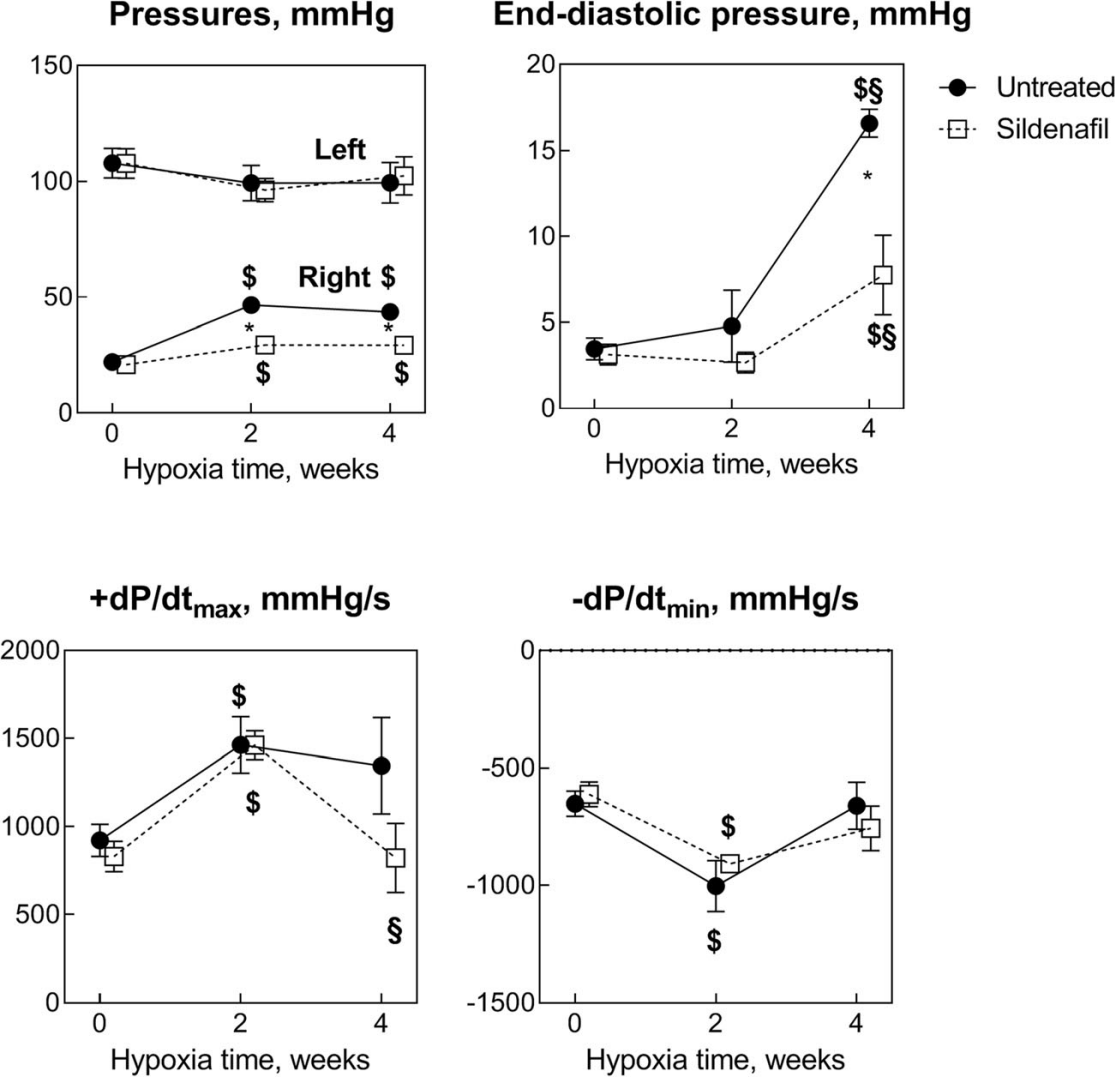
Hemodynamic parameters. Effects of various durations of hypoxia in the absence (filled circles) and in the presence (empty squares) of sildenafil (1.4 mg/kg/day). The vertical bars represent the SEM. Sildenafil data points are slightly nudged to the right to enable distinguishing overlapping symbols and error bars. $,§*P* < 0.05 (ANOVA and Bonferroni tests) with respect to *t*0 and *t*2, respectively. *P < 0.05 (unpaired Student’s *t* test) between control and sildenafil groups for each time point

Figure 2 shows several hemodynamic parameters. The LV systolic pressure remained unaffected by hypoxia (*P* = NS vs. *t*0) and sildenafil (*P* = NS). By contrast, the RV systolic pressure nearly doubled at *t*2 in controls (*P* < 0.05 vs. *t*0), indicative of PH, with no change at *t*4. In sildenafil rats at *t*2, the raise in RV systolic pressure was markedly less (+ 41% vs. + 108% in controls, *P* = 0.0014) and was maintained at *t*4 (+ 40% vs. + 97%, *P* = 0.002). The end-diastolic pressure did not change appreciably at *t*2, but it increased significantly at *t*4. The increase was + 377% (*P* < 0.0001) and + 147% (*P* = 0.02) in control and sildenafil rats, respectively, with a highly significant difference between the two groups (*P* = 0.007). By contrast, + dP/dt_max_ and − dP/dt_max_ were significantly increased + 50–70% at *t*2 in both groups with no significant difference. At *t*4, sildenafil blunted the increase in + dP/dt_max_ vs. no significant effects in − dP/dt_max_.

**Fig. 3:**
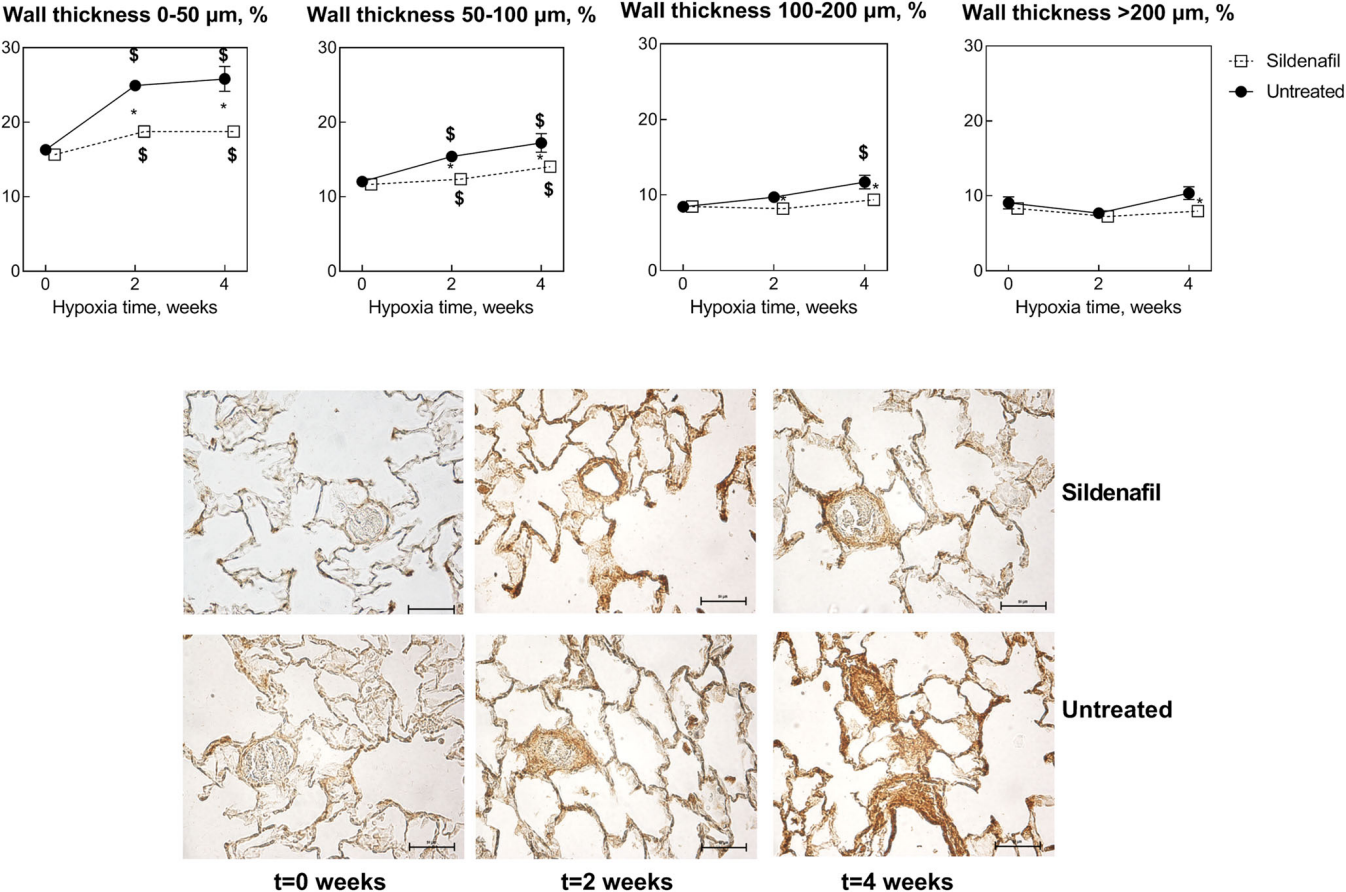
Effects of various durations of hypoxia in the absence (filled circles) and in the presence (empty squares) of sildenafil (1.4 mg/kg/day). The vertical bars represent the SEM. Sildenafil data points are slightly nudged to the right to enable distinguishing overlapping symbols and error bars. $,§*P* < 0.05 (ANOVA and Bonferroni tests) with respect to *t*0 and *t*2, respectively. *P < 0.05 (unpaired Student’s *t* test) between control and sildenafil groups for each time point. The microphotographs show representative examples of the Masson’s staining to evaluate lung tissue fibrosis in the various cases considered in this study

Figure 3 shows the frequency of lung vessels belonging to four categories of wall thickness, arbitrarily divided into small (0–50 μm), medium (50–100 μm), large (100–200 μm), and very large (> 200 μm). Because data from right lungs are indistinguishable from those from left lungs (not shown), for clarity, we report only data from left lungs. It appears that the effect of 2-week hypoxia was much pronounced for small vessels and progressively diminished for increasing vessel diameter. For example, the number of vessels in the 0–50 μm range increased by 35% vs. 11% for 50–100 μm, and near zero for larger vessels. As a result, the effect of sildenafil was more marked in small than in large vessels, 13% in the 0–50 μm range (*P* < 0.0001 vs. control) and virtually zero for larger vessels. In no case, however, was there a difference between *t*2 and *t*4, indicating a stable situation.

**Fig. 4:**
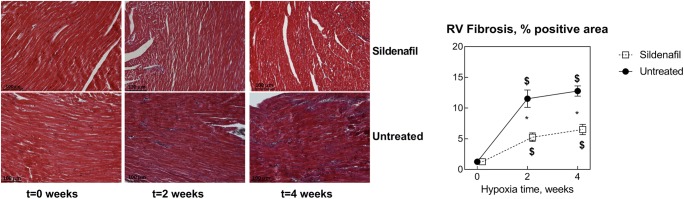
Effects of various durations of hypoxia in the absence (filled circles) and in the presence (empty squares) of sildenafil (1.4 mg/kg/day) on RV cardiac fibrosis. The vertical bars represent the SEM. Sildenafil data points are slightly nudged to the right to enable distinguishing overlapping symbols and error bars. $,§*P* < 0.05 (ANOVA and Bonferroni tests) with respect to *t*0 and *t*2, respectively. *P < 0.05 (unpaired Student’s *t* test) between control and sildenafil groups for each time point. The microphotographs show representative examples of the Masson’s staining to evaluate tissue fibrosis in the various cases considered in this study

Figure 4 shows indexes of RV fibrosis obtained as explained in the “Methods” section. While hypoxia increased fibrosis (*P* < 0.0001), the increase was markedly more pronounced in control vs. sildenafil rats (*P* = 0.007 and 0.0005 at *t*2 and *t*4, respectively), indicating that sildenafil has a strong anti-fibrotic effect in this model.

**Fig. 5:**
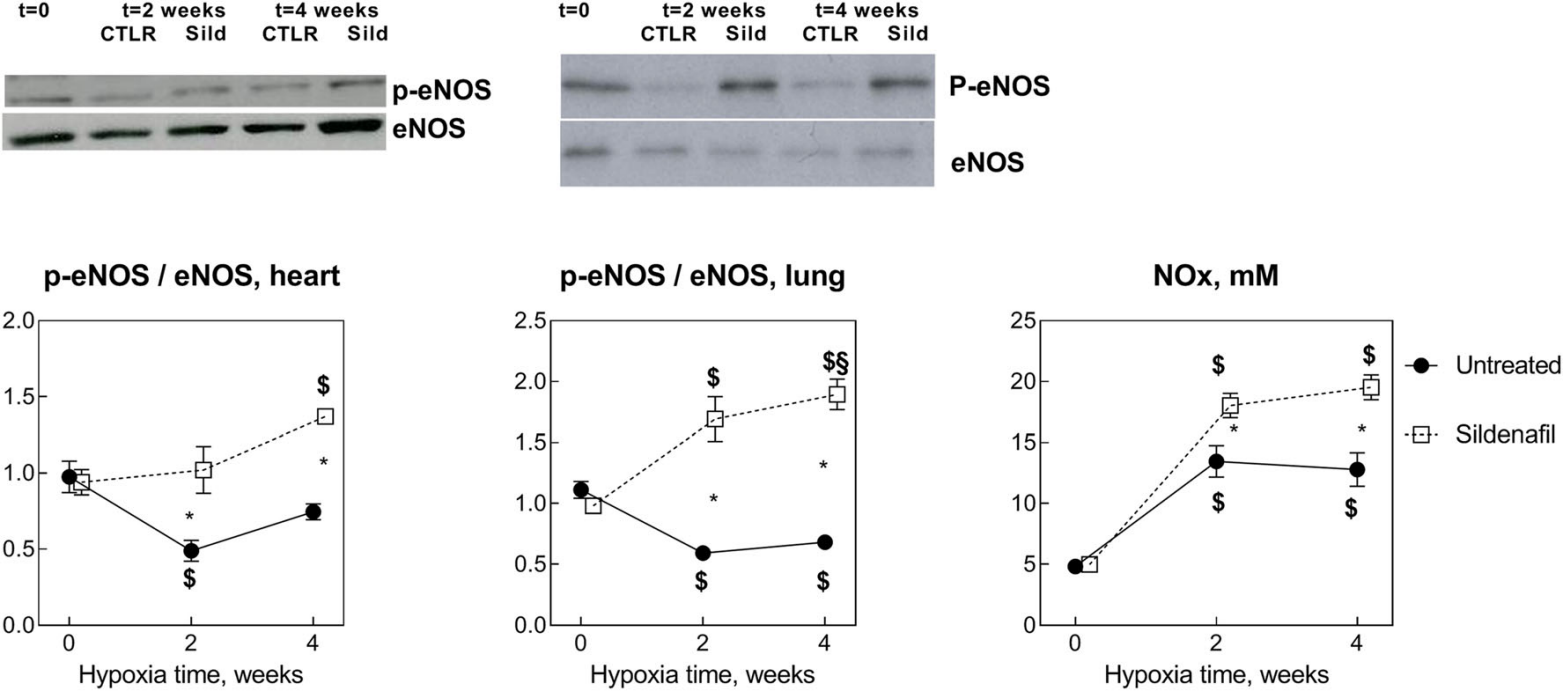
Effects of various durations of hypoxia in the absence (filled circles) and in the presence (empty squares) of sildenafil (1.4 mg/kg/day). The vertical bars represent the SEM. Sildenafil data points are slightly nudged to the right to enable distinguishing overlapping symbols and error bars. $,§*P* < 0.05 (ANOVA and Bonferroni tests) with respect to *t*0 and *t*2, respectively. *P < 0.05 (unpaired Student’s *t* test) between control and sildenafil groups for each time point. Representative Western blots are shown

Figure 5 shows that the p-eNOS/eNOS ratio highlights the activation of endothelium NO-producing enzymes in the heart and in the lung. In both cases, 2-week hypoxia decreased the p-eNOS/eNOS ratio in control (*P* < 0.05), as opposite to constant and increased p-eNOS/eNOS ratio in sildenafil-treated hearts (*P* = 0.01) and lungs (*P* < 0.0001), respectively. At *t*4, the situation remained stable, highlighting a protective effect of sildenafil in both tissues independent of hypoxia duration.

Plasma NOx concentration marks the blood NO storage level. Two-week hypoxia increased this marker in both control and sildenafil rats (*P* < 0.05 vs. *t*0), but in sildenafil-treated rats, this effect was higher (*P* = 0.007). The situation did not change appreciably at *t*4.

**Fig. 6:**
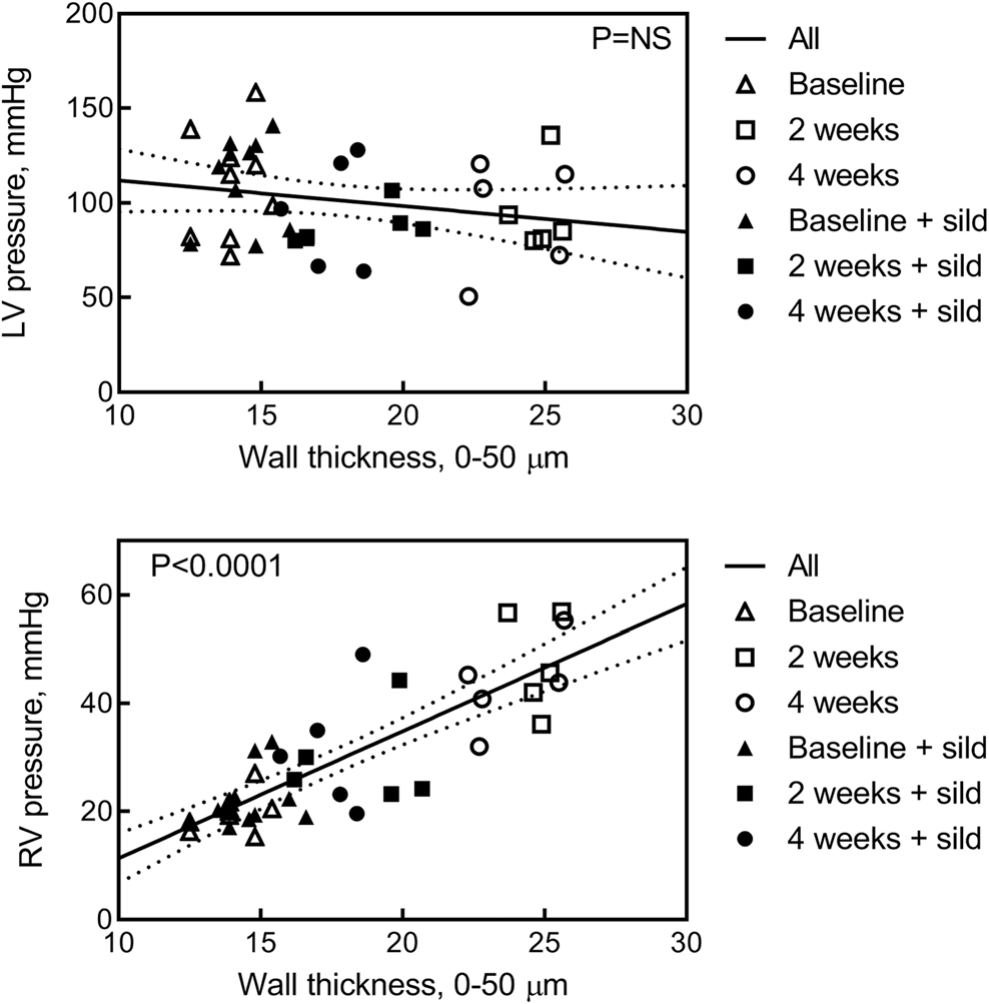
Relationship between the frequency of small diameter vessels (0–50 μm) and the pressure developed by the left (upper panel) and right (lower panel) ventricle for each experimental data point. Empty and filled symbols refer to rats treated with placebo and sildenafil, respectively. Triangles, squares, and circles represent data taken at *t*0, *t*2, and *t*4, respectively. The best-fit lines and the 95% confidence limits referring to all available data points are shown. The values of the slope are − 1.357 ± 0.9287 and + 2.352 ± 0.2563 for the left and right ventricle, respectively (*P* = NS and *P* < 0.0001 vs. zero)

To further assess the effects observed above, Fig. 6 shows the relationship between the frequency of small vessels (0–50 μm) and the pressure developed by LV (upper panel) and RV (lower panel). As far as the LV is concerned, the slope of the best-fit line (− 1.357 ± 0.9287) did not differ from zero (*P* = NS). However, the best-fit slope increased markedly to + 2.352 ± 0.2563 (*P* < 0.0001 vs. zero) in the RV. Of interest, the relationship between the RV systolic pressure and the small vessels frequency was independent of hypoxia duration, indicating that the treatment with sildenafil could reduce the RV systolic pressure for both time durations.

## Discussion

In our observations, 2- and 4-week duration of hypoxia induced relevant non-lethal cell and molecular alterations. As expected, hypoxia impaired the homeostasis and increased blood [Hb] and NOx, and increased the degree of RV hypertrophy, the extent of pulmonary angiogenesis, the total number of small vessels in the lungs, and the values of RV systolic pressures, without any direct or secondary influence on LV pressures. Most, but not all of these changes, occur within 2 weeks and are essentially maintained unchanged at the 4-week interval. However, some of the variables investigated, including blood [Hb] and RV hypertrophy, continue to change with the extension of the duration of hypoxia from 2 to 4 weeks. Whereas augmenting [Hb] reflects a situation of ongoing hypoxia without complete adaptation, increased RV hypertrophy reflects the long-term morphological effects of pro-angiogenic and proliferative factors engaged during the first 2 weeks of hypoxia. Thus, although adaptation seems to be a process not yet completely accomplished after 4-week hypoxia, most of the myocardial and pulmonary responses to hypoxia are independent from the total process of adaptation.

NO handling is of critical relevance because NO controls myriads of cell/molecular pathways relevant for heart and lung viability. For example, RV hypertrophy without lung hypertrophy, as well as the raise of RV pressure, without effects on LV pressure, has been associated with increased e-NOS phosphorylation in both hearts and lungs, and increased plasma NOx levels [13]. Remarkable blunting of all these features through PDE5 inhibition by sildenafil represents a solid proof-of-concept for the employment of this drug to alleviate symptoms in several cardiopulmonary diseases including PH [4] and to correct hypoxia-induced RV hypertrophy [1]. This scenario reflects a situation where sildenafil-induced increase in cGMP levels upregulates eNOS phosphorylation [15–20], and hence plasma NOx, which translates into cardiomyocyte apoptosis mitigation. In addition to its usefulness to treat heart failure without remarkable adverse effects [21], sildenafil may also have effects not only necessarily linked to vasodilation, as for example increased recruitment of bone marrow-derived c-kit+ cells with amelioration of pulmonary hemodynamic [14]. These mechanisms, however, have been studied within a single 2-week hypoxia situation, and there are no clues to ascertain whether such mechanisms reflect a transient situation or remain constant overtime [1]. In this study, we demonstrate that most of the underlying mechanisms are essentially unaffected by the duration of hypoxia and thus reflect a stable condition, independent from hypoxia adaptation. This is supported by the observation that the values of the pulmonary artery pressures in healthy highlanders increase in correlation with altitude and with the degree of exercise, and are related with a delayed postnatal remodeling of the distal pulmonary arterial branches [22].

The entity of the distribution of the vessels within the lungs is markedly depending on the diameter of the vessels. While the size of the small pulmonary vessels is affected by the degree of hypoxia and by sildenafil administration, the effects of both hypoxia and sildenafil are progressively diminishing in correlation with the size of the vessels. If we accept that the size of the vessel is associated with their degree of maturation, then this effect may be attributed to their maturation process. Although there is still disagreement about the molecular trigger of such process, the degree of phosphorylation of eNOS is most probably an important mediator; its persistent increase after 2 and 4 weeks of hypoxia in both the myocardial and pulmonary tissues of the sildenafil-treated rats reflects the angiogenic stimulus caused by hypoxia and the persistent correction of this stimulus by sildenafil.

The response to sildenafil, evident at both t2 and t4, can be considered to be due to the combination of the indirect effects on the RV function as a consequence of reduced afterload secondary to reduced pulmonary vascular resistance, as well as of the direct effect on the RV diastolic function. Whereas the effect of the afterload reduction was already well established [23], the capability of sildenafil to improve RV diastolic function was also previously reported in the presence of fixed afterload increase, such as that obtained by pulmonary artery banding [24]. This double action of sildenafil was confirmed by our observations, with the RV hemodynamics improved by sildenafil despite the presence of RV fibrosis at both *t*2 and *t*4.

With regard to the correlation between RV function and fibrosis, topic of controversies in the recent literature, it is important to evaluate presence, degree and distribution of fibrosis in the RV, as well as the duration of fibrosis. All these can influence not only RV systolic function, as generally studied and reported, but also diastolic function. Our observations, for instance, have proven the positive effects of sildenafil on RV diastolic function, regardless of the associated effects of the pulmonary vascular resistance, because the degree and distribution of RV fibrosis remained unchanged after 2 and 4 weeks. Masson staining is a reliable procedure to evaluate RV fibrosis [25]. The results shown in Figs. 2 and 4 are in essential agreement with a recent report that, although RV fibrosis is clearly linked to RV dysfunction, reduction of RV fibrosis does not directly improve cardiac function [26].

### Limits of the study

As a consequence of the design of our study, the treatment with sildenafil was started simultaneously with the induction of chronic hypoxia, and we are fully aware of the potential criticism of missing the evaluation of the potential effects of sildenafil after establishing chronic hypoxia. On the other hand, the effects of sildenafil on pulmonary hypertension per se are well acknowledged. Moreover, the RV diastolic pressures, which could provide useful information in order to correlate the values of RV end-diastolic pressure with the degree of RV hypertrophy and/or of PH, were not recorded in this study because of logistic reasons.

In this study, sildenafil was not compared with other pulmonary vasodilator drugs which recently have appeared on the market, because our aim was to specifically evaluate the effects of sildenafil, without adding other confounding factors.

## Conclusions

Sildenafil provides an efficient strategy for the management of RV hypertrophy, PH, and pulmonary remodeling, thanks to its effects on the pulmonary vasculature, facilitating improvement of the RV function, irrespectively of hypoxia adaptation. This finding suggests that sildefanil potentially could be used in the management of clinical settings characterized by pulmonary hypertension complicated by subsequent chronic hypoxia, as in PH secondary to congenital heart defects or acquired lung issues with chronic obstructive disease. Prospective randomized clinical trials are required to verify the applicability of this therapeutic strategy in patients.
